# Evolution of tigecycline resistance in *Klebsiella pneumoniae* in a single patient

**DOI:** 10.4103/0256-4947.67087

**Published:** 2010

**Authors:** Nada S. Al-Qadheeb, Sahar Althawadi, Abdulaziz Alkhalaf, Suleiman Hosaini, Abdulrahman A. Alrajhi

**Affiliations:** a From the Pharmacy Services Division, King Faisal Specialist Hospital and Research Centre, Riyadh, Saudi Arabia; b From the Section of Microbiology, Department of Pathology and Laboratory Medicine, King Faisal Specialist Hospital and Research Centre, Riyadh, Saudi Arabia; c From the Section of Infectious Diseases, Department of Medicine, King Faisal Specialist Hospital and Research Centre, Riyadh, Saudi Arabia; d From the Department of Adult Critical Care Medicine, King Faisal Specialist Hospital and Research Centre, Riyadh, Saudi Arabia

## Abstract

Carbapenemase-producing *Klebsiella pneumoniae* infections carry serious clinical and infection-control implications. Isolates possessing such hydrolyzing enzymes have been described in the United States and around the world. Besides being resistant to carbapenems, they usually confer resistance to fluoroquinolones, piperacillin-tazobactam, and extended-spectrum cephalosporins. Tigecycline demonstrates in vitro activity against these organisms, but reported resistance raises concern about tigecycline use for these infections. We describe a carbapenemase-producing *K pneumoniae* evolving resistance to tigecycline in a 75-year-old male after a prolonged stay in a critical care unit.

The prevalence of extended-spectrum β-lactamase (ESBL)-producing *Klebsiella pneumoniae* has been rising in the United States, approaching 50% of isolates in some regions.^1^ Similarly, there are several cases reported in Saudi Arabi. 2–10 The presence of an ESBL is a useful marker of the multidrug-resistant (MDR) phenotype. When such high rates of ESBL-producing organisms are encountered, carbapenems (imipenem, meropenem and ertapenem) become an increasingly important therapeutic option. Carbapenems have an especially broad spectrum of activity and remain effective against MDR gram-negative bacteria. However, the resistance to these agents has created a treatment challenge. In the United States, carbapenem resistance has been largely attributed to expression of a class C cephalosporinase and loss of outer membrane porins in isolates of *Acinetobacter baumannii, Pseudomonas aeruginosa* and rarely *K pneumoniae*. Carbapenem-hydrolyzing β-lactamases have been rarely recovered in *K pneumoniae*. Of concern, isolates possessing *K pneumoniae* carbapenemase (KPC) enzymes KPC-1, KPC-2 and KPC-3, class-A, have been identified in the United States and the rest of the world.^11^ These hydrolyzing enzymes are carried on transposable elements on plasmids, which facilitates the transmission of carbapenem resistance among different strains.^1^ Outbreaks have been described recently in some areas in the United States.^12^ The first KPC-producing *K pneumoniae* isolates were described in North Carolina in 2001 and subsequently in Baltimore, Maryland in 2003.^1^,^13^ Detection from other countries was reported in 2005. Isolates were reported in China, South America, Greece, the United Kingdom, Sweden, Germany and many other countries, but we are not aware of any cases reported in Saudi Arabia.^11^ Besides being resistant to carbapenems, KPC-producing strains usually confer resistance to fluoroquinolones, piperacillin-tazobactam and extended-spectrum cephalosporins.^1^,^14^ In vitro testing suggests that 50% of KPC-producing *K pneumoniae* isolates remain susceptible to aminoglycosides, and more than 90% are susceptible to polymyxins and colistin (polymyxin E). In the absence of other mechanisms, the KPC carbapenemase may not confer resistance to carbapenems but only reduce susceptibility, and some phenotypic tests may suggest KPC producers are ESBL producers.^15^ The presence of KPC-producing isolates may be clinically unrecognized because several factors can complicate their detection. A study published by Gasink et al demonstrated that severity of illness, prior fluoroquinolone use and prior extended-spectrum cephalosporin use are risk factors for isolation of KPC-producing *K pneumoniae*.^16^

Therapeutic options for highly resistant carbapenemase-producing organisms are limited. Polymyxin E and tigecycline both seem to have reliable efficacy, although resistance to both agents is already emerging. Tigecycline has promising in vitro activity against KPC-producing strains, but the clinical experience with this agent is still limited. Low-grade tigecycline resistance in members of the family Enterobacteriaceae has been reported and attributed to efflux pump mechanisms.^17^ The drug has extensive tissue penetration but relatively low peak serum concentration, which limits its use in the treatment of bloodstream infections. We describe the evolution of tigecycline resistance in a single patient with nosocomial pneumonia caused by a KPC-producing strain of *K pneumoniae*.

## CASE

A 75-year-old male known to have hypertension, chronic kidney disease, chronic obstructive airway disease (not known to be colonized), HIV infection on antiretroviral therapy and undetectable virus, a CD4+ T-lymphocyte count of 140 cells/mm^3^ and ischemic cardiomyopathy with an ejection fraction of 45% presented to the emergency room complaining of shortness of breath at rest for 1 week, productive cough and orthopnea. He was admitted to the intensive care unit (ICU) for decompensated heart failure and severe community-acquired pneumonia requiring intubation. He had acquired immunodeficiency syndrome (AIDS), a history of central nervous system toxoplasmosis, cytomegalovirus (CMV) colitis and pulmonary nocardiosis, but no active opportunistic infection. On admission, he was started empirically on meropenem and azithromycin. Although respiratory cultures showed no growth, the patient did better and was extubated and transferred to the floor several days later.

Recuperation was very slow. A month later, he had vomiting, respiratory distress, a temperature of 38.5°C and hypotension. The peripheral white blood cell count was 16 000 cells/mm^3^, and chest x-ray showed right lower lobe infiltrate. The impression was respiratory failure and septic shock secondary to aspiration pneumonia complicated by acute and chronic renal failure requiring transfer to the ICU, intubation, vasopressor support and the initiation of renal replacement therapy. Cultures were obtained, and patient was started empirically on vancomycin and piperacillin-tazobactam. In the ICU, the patient remained febrile on vasopressors. Cultures from the tracheal aspirates were positive for yeast and a few mixed gram-negative organisms. Because of lack of response to initial antibiotics, piperacillin-tazobactam was replaced with meropenem and colistin. On ICU day 4, *K pneumoniae* was recovered from the cultures of tracheal aspirates (isolate number 1). It was MDR, including carbapenems and colistin, but was susceptible to tigecycline. On ICU day 8, the patient improved hemodynamically, with resolution of fever and a normalized white blood cell count. Vancomycin and colistin were discontinued and meropenem was continued despite culture showing an MDR organism. On ICU day 13, the patient became febrile again, with a temperature of 38.6°C. Respiratory culture showed *K pneumoniae*, which also was isolated from the tip of an arterial line but not from blood. He was started on tigecycline, which was continued for 14 days. The patient responded favorably with fever resolution and improved oxygenation. Cultures from the tracheal aspirates 3 weeks later grew *K pneumoniae* again (isolate number 2), but this time it was intermediate to tigecycline. It was also grown from urine at the same time. Tigecycline was resumed along with meropenem for *Pseudomonas* from urine and respiratory secretions. Over the next few weeks, the patient’s condition worsened despite all supportive measures. Other gram-negative organisms were also isolated from respiratory secretions and urine (Proteus, *Pseudomonas*). *K pneumoniae* sensitive to carbapenems was isolated from respiratory secretions, urine and the catheter tip. It was not tested for tigecycline. Eventually, while he was receiving tigecycline, *K pneumoniae* was isolated from blood, two strains—one was resistant to tigecycline and all other antibiotics except aminoglycosides (isolate number 3), and the other strain was intermediate to tigecycline. The patient died a few days later. The last blood culture grew *Pseudomonas*.

## DISCUSSION

We describe a critically ill patient who received tigecycline for the treatment of nosocomial pneumonia caused by KPC-producing *K pneumoniae*. Based on antibiotic susceptibility testing profiles, the patient had several *K pneumoniae* isolated from different sources repeatedly (tracheal aspirate, blood, vascular catheter tip and urine). These isolates were tested using a custom gram-negative card for the automated system Vitek-2 (bioMérieux) that included tigecycline. As a policy in the Microbiology Laboratory, all Enterobacteriaceae isolates that are resistant to extended-spectrum cephalosporins (cefotaxime, ceftazidime or ceftriaxone) or have carbapenem (meropenem, imipenem and ertapenem) minimum inhibitory concentrations (MIC) of 2 or 4 μg/mL are screened for KPC-type carbapenemases. Fosfomycin, a broad-spectrum antibiotic, is indicated by Clinical Laboratory Standard Institute (CLSI) for testing against *Escherichia coli* urinary tract isolates. Therefore, our isolates were not tested against it.^18^ The meropenem and imipenem Vitek-2 MIC results were confirmed by E-test, and CLSI breakpoints were used for MIC interpretations.^19^ There are no CLSI breakpoints for tigecycline and Enterobacteriaceae, but US Food and Drug Administration breakpoints from pharmaceutical product labeling data were used (≤2, sensitive; 4, intermediate; ≥8, resistant).

**Figure 1:**
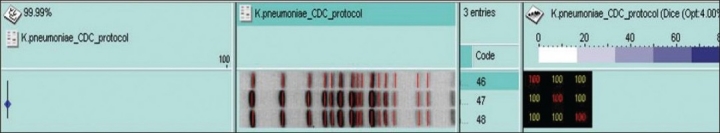
Pulsed-field gel electrophoresis of the three isolates of *K pneumoniae*. Computer analysis of *Xbal* digested *K pneumoniae* isolates, using BioNumerics software (Applied Maths).

Molecular testing for blakpc genes is not available in our facility to confirm the presence of KPC production. Instead, the phenotypic testing method, which is modified Hodge test (a carbapenem-inactivating assay), was used. Utilizing this phenotypic test on all Enterobacteriaceae that have elevated MIC to carbapenems is a reliable screening method for KPC production. Pulsed-field gel electrophoresis (PFGE) was performed on the first three isolates of *K pneumoniae* as described previously.^20^ Results were analyzed by computer analysis using BioNumerics software (Applied Maths). PFGE result showed that the 3 isolates were genetically indistinguishable and represented one predominant strain (Figure 1). Unfortunately, isolate number 4 was not saved for further testing.

Hawser et al showed that the activity of tigecycline against KPC-negative and KPC-positive isolates in 86 of 2645 clinical isolates collected from 10 countries during 2005 to 2008 was similar. One third (29/86) of the isolates were ESBL-positive. Polymerase chain reaction screening of the 86 isolates revealed that 26 (30%) isolates were KPC-positive and the remaining isolates were KPC-negative. The MIC90 values (minimum inhibitory concentrations for 90% of the isolates) against KPC-negative isolates were 2, >16, 16 and 16 μg/mL for tigecycline, ertapenem, imipenem and meropenem, respectively. This corresponded to susceptibilities of 96.6%, 63.4%, 80.1% and 75% for the four agents, respectively. As expected, KPC-positive isolates exhibited reduced susceptibility to the carbapenems, with MIC90 values of >16, 32 and >32 μg/mL for ertapenem, imipenem and meropenem, respectively. The corresponding susceptibilities were 3.8%, 19.2% and 19.2%. By contrast, the activity of tigecycline against KPC-negative and KPC-positive isolates was similar. Against KPC-positive isolates, tigecycline exhibited MIC90 of 2 μg/mL (100% of KPC-positive isolates susceptible). No KPC-positive isolate had an imipenem MIC value ≤1 μg/mL, whilst only 3 isolates had MICs ≤1 μg/mL for either ertapenem or meropenem.^21^

Anthony et al reported on the evolution of tigecycline resistance in *A baumannii* and other Enterobacteriaceae, including *K pneumoniae*, during therapy, which raises concern about the routine use of tigecycline in these infections until more solid data are available.^22^ We have demonstrated in this case that the same strain of *K pneumoniae* as confirmed by PFGE in the same patient evolved from being susceptible to intermediate to resistant to tigecycline. The general condition of the patient, tracheostomy and ventilation contributed to the failure to eradicate this organism. We have used amikacin for several days concurrently with tigecycline, but that was not effective either. We believe that until we gain more experience in using tigecycline for *K pneumoniae* and until its role is studied better, tigecycline should be used cautiously for MDR *K pneumoniae* and not solely. The addition of an aminoglycoside to which *K pneumoniae* is susceptible may be a required measure.

In brief, the emergence of KPC-producing *K pneumoniae* strains throughout the entire world reinforces the importance of antibiotic stewardship and strict infection-control practices. Healthcare providers are encouraged to follow the Center for Disease Control and the Healthcare Infection Control Practices Advisory Committee recommendations for the control of carbapenemase-producing Enterobacteriaceae in healthcare facilities. They should help in establishing a protocol, in conjunction with CLSI guidelines, to detect nonsusceptibility and carbapenemase production in Enterobacteriaceae and immediately alert epidemiology and infection-control staff members if identified.^23^
